# Comparative Assessment of Morphometry, Morphology, and Maturation Capacity of Vitrified Cattle Oocytes in Different Media

**DOI:** 10.3390/vetsci12050461

**Published:** 2025-05-12

**Authors:** Maleke Dimpho Sebopela, Ntuthuko Raphael Mkhize, Mamonene Angelinah Thema, Masindi Lottus Mphaphathi

**Affiliations:** 1 Agricultural Research Council, Animal Production, Germplasm Conservation and Reproductive Biotechnologies, Private Bag X2, Pretoria 0062, South Africa; sebopela08@gmail.com (M.D.S.); mamonenethema@gmail.com (M.A.T.); 2 School of Agricultural, Earth and Environmental Sciences, University of KwaZulu-Natal, Private Bag X01, Scottsville 3209, South Africa; mkhizen31@ukzn.ac.za

**Keywords:** cryopreservation, immature and mature oocytes, polar body

## Abstract

This study aimed to compare the morphometry, morphology, and maturation capacity of cattle oocytes subjected to vitrification using different vitrification and maturation media. In Experiment 1, a total of 900 oocytes were divided into three groups: (1) matured before vitrification, (2) non-vitrified, and (3) vitrified as immature oocytes using the straw vitrification method. Morphometric parameters, including oocyte diameter, ooplasm, zona pellucida width (ZPW), granulosa cell width (GRSW), and zona pellucida-granulosa cell width (ZP GRSW), were measured (µm) before and after cryopreservation. In Experiment 2, the maturation capacity of three in vitro maturation (IVM) media (VitroMat-Protect™, BO-IVM™, and TCM199) was evaluated based on cumulus–oocyte complex (COC) expansion and polar body (PB) extrusion. Morphological abnormalities such as fragmented polar bodies (FPBs), large vacuoles (LVs), degenerated oocytes (DOs), and cracked cytoplasm (CC) were recorded. While vitrification did not significantly affect the oocyte diameter, ooplasm, or ZPW, it significantly reduced the GRSW and ZP GRSW. BO-IVM™ supported the highest COC expansion rate, while TCM199 had the lowest. Among vitrified oocytes, the highest PB extrusion rates were observed in BO-IVM^TM^ (35.14 ± 5.01) and Vitromat-Protect^TM^ (24.60 ± 5.67) as compared to TCM199 (18.44 ± 8.00; *p* < 0.05). Oocytes with higher CC rates were observed in VitroMat-Protect™ (24.50 ± 10.53) and BO-IVM™ (31.42 ± 7.32) as compared to TCM199 (18.70 ± 7.04). In conclusion, the vitrification process affects the granulosa cells in both vitrified immature and mature oocytes. BO-IVM^TM^ and VitroMat-Protect^TM^ supported better oocyte maturation than TCM199, although vitrification increased FPB and CC rates.

## Simple Summary

To date, oocyte cryopreservation, particularly vitrification, is the most effective and affordable method for preserving the female germplasm across species. Various media contain different combinations and concentrations of cryoprotectants for this technique. However, the cryopreservation process affects the oocyte morphology, compromising its viability and developmental potential. Selecting accurate vitrification solutions and warming and maturation media is crucial for improving fertilization outcomes. Selecting optimal media effectively reduces the impact of cryo-induced damage. Therefore, it is essential to use optimized vitrification solutions to enhance preservation outcomes. The primary goal of cryopreservation is to preserve the structural and functional integrity of oocytes during both the freezing and thawing processes. Oocyte quality is essential for successful embryo development and presents a major challenge in animal research technology. In vitro maturation of the oocytes, which involves maturation of both the nucleus and cytoplasm, is one of the key steps of in vitro embryonic production. Given the importance of media in clinical outcomes, well-designed protocols are needed to improve oocyte progression from vitrified oocytes.

## 1. Introduction

Successful cryopreservation of cattle oocytes would facilitate the management of the timing of in vitro maturation (IVM), fertilization (IVF), and culture (IVC). Cryobiology focuses on improving vitrification outcomes for both immature and mature oocytes and is still challenging. It is crucial to acknowledge that the current efficiency of in vitro embryo production (IVEP) significantly decreases by approximately 50% when utilizing vitrified cattle oocytes [1]. As a result, the practical application of this technique remains at an experimental stage [2]. To date, oocyte cryopreservation is the most promising and cost-effective option for storing the female germplasm, and vitrification is the method of choice for cryopreservation of oocytes in several species [3]. Cryoprotectants are organic solutes that protect cellular organelles during cryopreservation [4]. However, they can be toxic and disrupt the cytoskeletal system, leading to osmotic damage. Cattle oocyte vitrification can be achieved using cryoprotectant media, such as dimethyl sulfoxide or propanediol, combined with ethylene glycol or glycol alone. Combining cryoprotectants rather than a single solute may provide certain advantages, potentially improving oocyte survival and developmental outcomes [5,6]. Cryopreservation can alter oocyte morphology, which may reduce viability and developmental competence. Therefore, it is essential to use optimized vitrification solutions to enhance preservation outcomes.

The primary goal of cryopreservation is to preserve the structural and functional integrity of oocytes during both the freezing and thawing processes. Oocyte quality is essential for successful embryo development and presents a major challenge in animal research technologies (ARTs) [7]. Therefore, the molecular assessment of oocyte quality (morphology and morphometry) is paramount in predicting IVF outcomes [8]. The evaluation of oocyte quality before cryopreservation can be achieved via the visualization of spindles in metaphase II oocytes [9] or by measuring the spindle size using a polarization microscope [10]. It can improve the prediction of the embryo development potential and pregnancy outcomes of in vitro fertilized and intracytoplasmic sperm injection cycles using frozen–thawed oocytes. Previous studies in cattle and goats have demonstrated a correlation between oocyte size and their developmental competence [11,12]. In bovine [13] and caprine livestock [14], a minimum oocyte diameter of 115 μm is necessary for full meiotic competence, and full developmental competence is acquired at 120 μm. Oocytes selected for IVM are retrieved from follicles at different stages of folliculogenesis; most, although meiotically competent, have not undergone all essential cytoplasmic changes to support further development [7]. Moreover, the selection criteria currently used are based on subjective morphological parameters; many surrounding cumulus cells, slightly expanded investment without dark areas, a large oocyte diameter (>120 microns), a dark cytoplasm, and the presence of a round and smooth first polar body (PB) have been associated with better competence, perivitelline space, and cytoplasm, and so these are analyzed by controversial microscopy [15].

The PB biopsy before oocyte cryopreservation can also predict euploid embryos and future pregnancy outcomes. Oocytes with the first euploid PB have more chance of forming blastocysts [16]. Maturation media, containing various nutrients, growth factors, and supplements, provide the necessary environment for oocytes to mature, fertilize, and develop into embryos [17]. The selection of maturation media is critical because it can directly affect oocyte quality, fertilization rates, embryo development, and overall success rates in ART [18]. Culture media should support the oocytes’ metabolic needs, maintain appropriate pH levels, provide osmotic stability, and promote the necessary signaling for successful fertilization and subsequent embryonic development [19]. Most media for IVEP require supplementation with various compounds before use in the IVF laboratory, which is time-consuming and increases the risk of batch inconsistencies. To meet the special needs of vitrified oocytes, several culture medium formulations have been created and improved [20]. These formulations often contain essential nutrients such as amino acids, vitamins, and energy sources that support oocyte maturation and embryonic development. Additionally, growth factors and hormones are frequently added to mimic the physiological conditions found in the female reproductive tract and enhance the developmental competence of vitrified oocytes.

Numerous studies have evaluated the effectiveness of different media in promoting the maturation, fertilization, and development competence of cattle immature oocytes [21,22]. These studies often assess parameters such as fertilization rates, embryo quality, blastocyst formation rates, and even subsequent pregnancy rates on non-vitrified and vitrified oocytes. Furthermore, several maturation media, such as tissue culture medium 199 (TCM 199, commonly used), Bracket, and Oliphant’s in vitro maturation (BO-IVM^TM^, Biosciences media), have been used before on oocyte maturation to improve IVM results and options mostly in non-vitrified oocytes. This study aims to introduce a newly developed vitrification and IVM medium (VitroMat-Protect™, ART Lab Solutions, Adelaide, Australia) to improve embryo development. It has not yet been used in South Africa for cattle oocyte cryopreservation in IVEP. This medium is internationally known for its capacity to improve oocyte cryotolerance, protect cumulus–oocyte complexes (COCs) from cellular stress, and provide additional support to the oocyte during maturation following cryopreservation. It also provides a good maturation quality and sustains the morphological structure of the oocytes. The optimization of culture conditions can lead to improved outcomes in ART, enabling more efficient livestock breeding and genetic improvement programs [23]. Ongoing research is improving the understanding of the specific requirements of vitrified oocytes and the best culture conditions for successful fertilization and development. Against that background, this study aimed to evaluate the morphometric parameters, morphology, and maturation capacity of immature, mature vitrified, and non-vitrified oocytes in different media.

## 2. Materials and Methods

### 2.1. Study Design

#### 2.1.1. Experiment I: Morphometric Evaluation of Immature and Mature Cattle Oocytes Pre and Post Cryopreservation

In this experiment, oocytes were divided into the following groups:

1. Control group (non-vitrified immature oocytes);
2. Vitrification of immature oocytes with ART Lab Solutions vitrification medium;
3. Vitrification of mature oocytes with ART Lab Solutions vitrification medium.

#### 2.1.2. Experiment II: Three Different Vitrification Protocols, Together with Their Respective Warming and Maturation Media, Were Evaluated

1. Control group (non-vitrified oocytes);
2. Immature oocytes were vitrified using TCM199-based vitrification medium (Gibco-Invitrogen Life Technologies, Grand Island, NY, USA) and subsequently subjected to IVM in TCM199 medium;
3. Immature oocytes were vitrified using ART Lab Solutions vitrification medium and subsequently subjected to IVM in VitroMat-Protect™ (ART Lab Solutions, Adelaide, Australia) medium;
4. Immature oocytes were vitrified using BO-VitriCool™ (Bioscience, Guangzhou, China) medium and subsequently subjected to IVM in BO-IVM™ (Bioscience, Guangzhou, China) medium.

### 2.2. Cattle Ovary Collection

Heterogeneous cattle ovaries of unknown reproductive status were collected from a local abattoir, and the ovaries were immediately transported to the GCRB laboratory in 0.9% saline solution (SABAX pour saline Adcock Ingram, Johannesburg, South Africa) in a thermos flask maintained at 37 °C. Upon arrival at the laboratory, the ovaries were washed with pre-warmed buffered saline to remove blood contamination. Then, the ovaries were placed in a 37 °C water bath (B. Owen Jones Ltd. Macdonald Adams & Company, Johannesburg, South Africa) until further processing.

### 2.3. Aspiration Method for Retrieving Cattle Oocytes

The aspiration method for oocyte retrieval was carried out using 10 mL disposable syringes (U-Life-Medical, Johannesburg, South Africa) and an 18-gauge sterile hypodermic needle (U-Life-Medical, RSA). The needle was pushed inside the ovaries and sucked out the follicular fluid of visible follicles. The recovered follicular fluid was searched for the recovered oocytes under the microscope (Olympus CX 23, Johannesburg, South Africa) at 80× magnification. Only recovered oocytes with full attachment of cumulus cells were considered for the experiment.

### 2.4. Experiment 1: Morphometric Evaluation of Immature and Mature Cattle Oocytes Pre and Post Cryopreservation

#### 2.4.1. Vitrification and Warming of Immature and Mature Cattle Oocytes

Three vitrification and warming protocols were used: The vitrification procedure was performed at room temperature. The retrieved oocytes were either non-vitrified or vitrified (immature and mature oocytes) using the conventional straw vitrification method, where they were exposed to different equilibration and vitrification solutions (VSs). The first group of immature and mature oocytes (*n* = 150) was exposed to VitroMat-Protect™ vitrification, holding medium (HM), and warming medium, the composition of which is indicated in Table 1. The mature oocytes were subjected to IVM (Section 2.5.1) pre cryopreservation. To evaluate the direct impact of the vitrification process on oocyte structural integrity, morphometric evaluation was carried out just on oocytes vitrified using the newly tested medium (ART Lab Solutions), thereby justifying its potential for further comparison against standard protocols.

The second group of oocytes (*n* = 150) was exposed to TCM 199 vitrification and warming medium, as indicated in Table 2.

The third group of oocytes (*n* = 150) was exposed to BO-VitriCool^TM^ and BO-VitriWarm^TM^, the composition of which is indicated in Table 3.

Prior to maturation, three vitrification and warming protocols were used as indicated in Table 1, Table 2 and Table 3; the vitrified oocytes consisted of a column of VS, an air bubble, a column of VS containing five to ten oocytes, another air bubble, and a final column of VS. The straws were pre-cooled horizontally on a Styrofoam rack, positioned 5 cm above liquid nitrogen (LN_2_) vapor for 5 min before being directly vitrified and immersed in LN_2_ (−196 °C). The oocytes’ frozen straws were loaded on the aluminum cryocane and stored inside the LN_2_ tank (−196 °C) until thawing. The non-vitrified oocytes were subjected to IVM for 22 h. These observations were repeated ten times.

#### 2.4.2. Thawing of the Immature and Mature Cattle Oocytes

The temperature was adjusted and maintained at 37 °C during the oocyte thawing. Frozen oocyte (conventional straw vitrification method) straws were removed from the LN_2_ tank (−196 °C) and exposed for 10 s in the air, then plunged into the thawing container. The straws were exposed to warm (37 °C) water for 1 min during the thawing. The oocyte straws were cut at both ends and emptied into the thawing solutions to remove the intracellular cryoprotectant. A first sterile four-well dish was filled with medium and used as follows: oocytes were washed thrice in M199 + 10% FBS to remove the intracellular cryoprotectant.

#### 2.4.3. Morphometric Evaluation of Immature and Mature Cattle Oocytes

**Figure 1:**
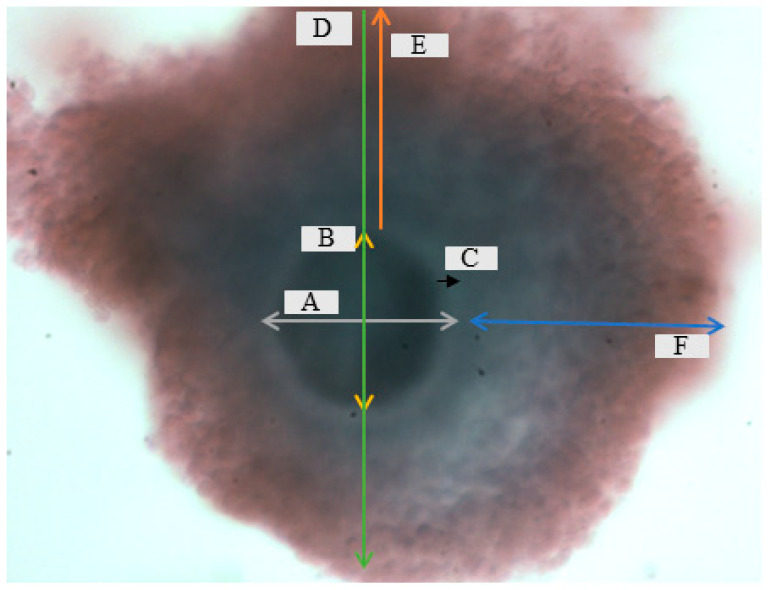
Dimensions that were measured during morphometry evaluation pre and post cryopreservation: A = oocyte, B = ooplasm (OPS), C = zona pellucida (ZP), D = oocyte granulosa cells (GRS), E = zona pellucida granulosa cells width (ZP GRSW), and F = granulosa cells width (GRSW) at ×10 magnification.

The vitrified drops were then transferred into a sterile four-well dish filled with a warming medium (Table 1). Following thawing, oocytes were fixed in 4% formaldehyde for morphometric analysis. The diameter of the oocyte, ooplasm (OPS), zona pellucida (ZP), granulosa cells (GRS), zona pellucida granulosa cell width (ZP GRSW), and granulosa cell width (GRSW) were measured (µm) with the aid of a microscope connected to a computer-assisted sperm analysis system at 10× magnification for both vitrified and non-vitrified oocytes (Figure 1). To calculate the average diameter of different parts of each oocyte compromising the whole oocyte, the ooplasm and ZP were calculated in 4 different parts. These observations were repeated ten times.

### 2.5. Experiment 2: To Compare the Maturation Rate and Morphological Characteristics of Oocytes Following Cryopreservation in Different Media

#### 2.5.1. In Vitro Maturation of Cattle Oocytes

The oocytes were washed six times before maturation: three times in modified Dulbecco’s phosphate-buffered saline (MDPBS) and three times in M199 + 10% fetal bovine serum (FBS). The four-well dishes (Thermo Scientific Nunclon Delta surface Sigma-Aldrich, Saint Louis, MO, USA) were used for IVM. Each well contained 500 µL of maturation medium (1: TCM199, containing medium 199 + 10% FBS supplemented with follicle stimulating hormone (FSH), luteinizing hormone, and estradiol hormone, sodium pyruvate, and antibiotics 2: BO-IVM^TM^, which is serum-free, supplemented with low glucose, gonadotrophic hormones, and gentamycin; 3: Vitromatprotect^TM^ containing 4 mg/mL bovine serum albumin and 100 mIU/mL of equivalents of FSH) covered with 250 µL mineral oil to prevent the maturation media from evaporating. We used 150 oocytes with a full or moderate attachment of cumulus cells per treatment, incubated at 38.5 °C under 5% carbon dioxide conditions. These observations were repeated ten times.

#### 2.5.2. Oocyte Cumulus Cells’ Expansion

Following 22 h in the maturation medium, the oocytes were checked for the expansion of cumulus cells as a sign of cytoplasmic maturation. Assessment of COC expansion was carried out as described by Maksura et al. [24] with some modifications. Briefly, COCs with uniformly granulated cytoplasm, not exhibiting degeneration (apoptotic or necrotic symptoms), and surrounded by compact layers of cumulus cells were classified as expanded COCs. All COCs other than expanded COCs, such as COCs without cumulus cell expansion (no observable sign of cumulus expansion), were classified as non-expanded COCs. The COC expansion of oocyte maturation was investigated by visualization of the expansion of COCs [25].

#### 2.5.3. Oocytes’ Polar Body Extrusion and Morphological Characteristics’ Evaluation

The oocytes were transferred to Eppendorf tubes (Micro, Centrifuge tube, New York, NY, USA) following 22 h of IVM. Oocytes were immersed in 200 μL M199 + 10% FBS and vortexed for 1 min and 30 s to remove the cumulus cells surrounding the oocytes. The extrusion of the oocyte PBs and development and the morphological parameters (fragmented polar bodies (FPBs), large vacuoles (LVs), degenerated oocytes (DOs), and cracked cytoplasm (CC)) of oocytes were examined under a micromanipulation microscope (Olympus, New York microscope Co., Hicksville, NY, USA) using the oosight Imaging USA system at 20× magnification. The number of oocytes with the presence and absence of PBs and the morphology of the oocytes were recorded. These observations were repeated ten times.

### 2.6. Statistical Analysis

The data were analyzed using the analysis of variance (ANOVA). The ANOVA analysis was performed using SAS version 9.4 statistical software. A significant level of *p* < 0.05 was used. Analysis of variance was used to test differences in the effect of post-thaw cryopreservation of oocytes in different maturation media. Treatment means were separated using Fisher’s protected t-test. The data were presented as mean ± standard deviation (S.D).

## 3. Results

### 3.1. Morphometric Evaluation of Immature and Mature Cattle Oocytes Pre and Post Cryopreservation

**Figure 2:**
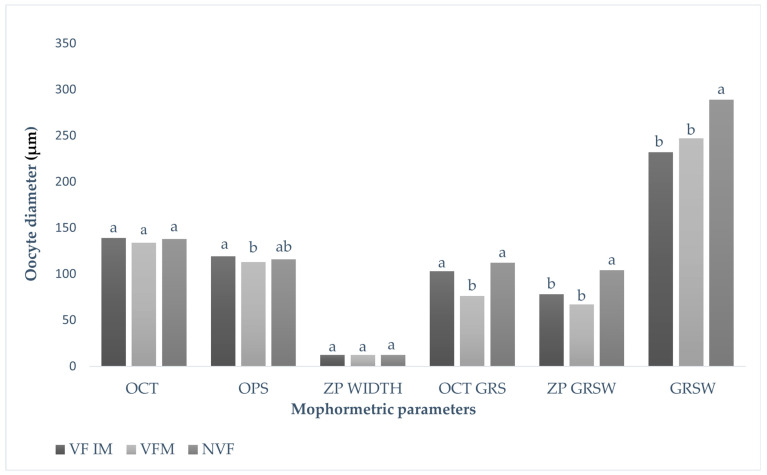
Oocyte morphometric analyses between non-vitrified (NVF), vitrified mature (VFM), and vitrified immature (VF IM) cattle oocytes: OCT = oocyte, OPS = ooplasm, ZP = zona pellucida, OCT GRS = oocyte granulosa cells, ZP GRSW = zona pellucida granulosa cells, and GRSW = granulosa cell width. a,b Values with different letters differ significantly (*p* < 0.05).

**Figure 3:**
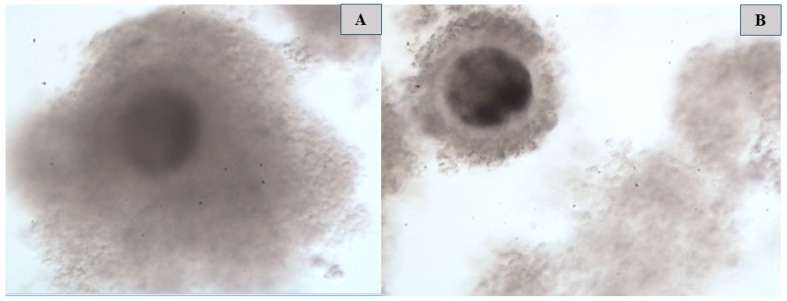
(**A**) = Morphological structure of non-vitrified oocytes (pre-cryopreservation), (**B**) = morphological structure of vitrified immature oocytes (post-cryopreservation) at ×10 magnification.

In this current study, the cytoskeletal structure of the oocytes was observed pre and post cryopreservation (Figure 2). The results showed no significant differences in oocyte ZP width between vitrified (immature and matured) and non-vitrified oocytes (Figure 3). However, a significant reduction was observed in both oocyte ZP GRSW and overall GRSW in vitrified groups compared to non-vitrified oocytes. Specifically, ZP GRSW was 67.56 ± 32.61 in vitrified matured oocytes and 78.46 ± 33.51 in vitrified immature oocytes, compared to 104.23 ± 27.21 in non-vitrified oocytes. Similarly, the overall GRSWs were 232.39 ± 50.34 and 247.25 ± 66.92 in vitrified mature and immature oocytes, respectively, versus 289.30 ± 42.19 in non-vitrified oocytes.

### 3.2. Comparison of the Maturation Rate and Morphological Characteristics of Oocytes Following Cryopreservation in Different Media

**Figure 4:**
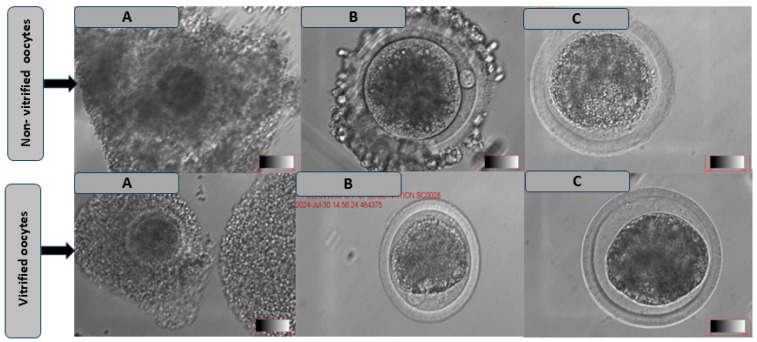
In vitro maturation of cattle non- and vitrified oocytes: (**A**) = cumulus oocytes complexes, (**B**) = polar body extrusion, and (**C**) = no polar body at ×10 magnification.

As shown in Table 4, the results demonstrated that oocytes vitrified and matured in BO-IVM^TM^ (52.28 ± 7.06) showed significantly higher cumulus oocyte expansion rates (Figure 4) as compared to those matured in Vitromat-protect^TM^ (34.00 ± 11.02) and TCM199 (28.70 ± 10.03; *p* < 0.05). In contrast, non-vitrified oocytes showed no significant differences in PB extrusion across the treatment groups. There were no significant differences in cumulus expansion between oocytes matured in Vitromat-protect^TM^ and those matured in TCM199. Among vitrified oocytes, the highest PB extrusion rates (Figure 4) were observed in BO-IVM^TM^ (35.14 ± 5.01) and Vitromat-protect^TM^ (24.60 ± 5.67), compared to TCM199 (18.44 ± 8.00; *p* < 0.05).

**Figure 5:**
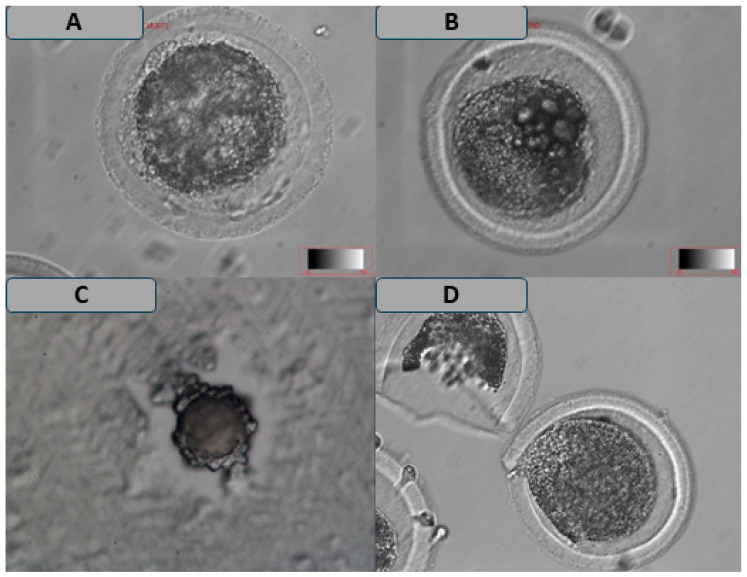
Various morphological abnormalities exhibited by the metaphase II stage were observed in vitrified oocytes ((**A**) = fragmented polar body, (**B**) = several large vacuoles, (**C**) = degenerated oocyte, and (**D**) = cracked cytoplasm) at ×10 magnification.

The results in Table 5 indicate a significant difference in the incidence of FPBs between vitrified oocytes matured in TCM199 (13.90 ± 4.54) and those matured in Vitromat-Protect^TM^ (19.30 ± 3.97). Furthermore, a higher proportion of vitrified oocytes exhibiting CC (Figure 5) was observed in Vitromat-Protect^TM^ (24.50 ± 10.53) and BO-IVM^TM^ (31.42 ± 7.32) as compared to those matured in TCM199 (18.70 ± 7.04; *p* < 0.05). In contrast, no significant difference was observed in the incidence of CC among non-vitrified oocytes matured in Vitromat-Protect^TM^, BO-IVM^TM^, and TCM199. Additionally, non-vitrified oocytes across the different maturation media exhibited intact cytoplasms (*p* > 0.05). No significant differences between LVs and DOs were observed among vitrified oocytes across all treatment groups. No significant differences were observed in the presence of vacuoles across all treatment groups in vitrified oocytes.

## 4. Discussion

This study evaluated the morphometry and morphology of the oocytes pre- and post-maturation following cryopreservation. No significant differences were found in the outer oocyte, ooplasm, and ZP width from non-vitrified, vitrified, and mature vitrified oocytes. Cattle oocytes with a diameter of 110 mm can achieve complete meiotic competence, while smaller oocytes have significantly less transcriptional activity, indicating that they are still in the growing phase [13]. Otoi et al. [26] indicated that meiotic competence was attained once oocytes reached 115 µm in diameter, while full developmental capacity was obtained when the diameter was at least 120 µm. In this study, the oocyte diameter measured 119 μm in vitrified immature oocytes and 116 μm in non-vitrified oocytes, suggesting that the vitrification process had no significant impact on oocyte diameter. Otoi et al. [26] reported that oocytes must measure at least 110 μm to reach the metaphase II stage. Other studies have indicated that nuclear maturation, such as that in pigs [27], buffalo [28], camels [29], and humans [30], and blastocyst production in cattle, were positively correlated with the oocyte diameter [26,31,32].

The same parameters employed in evaluating oocyte morphology in human oocytes by Lasiene et al. [30] include the appearance of the structure of the COCs, oocyte cytoplasm, perivitelline space, zona pellucida, polar body, and meiotic spindle. In this study, the reduction in GRS in vitrified immature and mature oocytes may indicate a decreased capacity to sustain effective communication between the oocyte and GRS following vitrification. Before maturation of the GRS, width produced a significant difference in the diameter of non-vitrified immature and vitrified matured oocytes. This is consistent with the findings by Brambillasca et al. [33], who reported that cryopreservation significantly affects the oocyte GRS, leading to altered communication between the oocyte and its surrounding cells. Additionally, after maturation, a highly significant difference decrease in GRS was observed. However, the significant differences observed in oocyte GRS and ZP GRSW between vitrified and non-vitrified oocytes could be attributed to the effects of vitrification or cryoprotectants on oocyte developmental competence and the surrounding GRS architecture. A study conducted by Mphaphathi et al. [34] indicated that an increase in cryoprotectants reduces the success of oocyte maturation in vitro.

In good physical condition in the intrafollicular environment, COCs surrounding the oocyte help with oocyte maturation [35]. The COC expansion is an essential indicator of oocyte quality. It is a key factor in oocyte fertilization potential, reflecting the functional interactions between the oocyte and its surrounding cumulus cells [36]. The COC expansion is regulated by factors such as hyaluronic acid synthesis, which is influenced by signaling pathways between the oocyte and cumulus cells [37]. In this study, oocytes vitrified and matured in BO-IVM^TM^ showed significantly higher COC expansion rates compared to those matured in VitroMat-protect^TM^ and TCM199, which suggests that BO-IVM^TM^ may provide a more conducive environment for oocyte maturation and subsequent COC expansion. In contrast, Vitromat-Protect^TM^ (newly tested media) and TCM199 (commonly used media) for oocyte maturation did not result in significantly different COC expansion in the vitrified oocytes compared to BO-IVM^TM^.

The PB extrusion is a critical marker of oocyte maturation because of the completion of meiosis, which is a necessary process for oocyte fertilization [38]. The PB extrusion was significantly higher for oocytes matured before vitrification in VitroMat-Protect^TM^ (67%), BO-IVM^TM^ (71%), and TCM199 (61%) than for oocytes vitrified after IVM in VitroMat-Protect^TM^ (24%), BO-IVM (35%), and TCM199 (18%). Similarly, Fasano et al. [39] showed that the IVM procedure is more efficient when it is performed before oocyte vitrification. Additionally, no significant differences were observed in PB extrusion rates between non-vitrified oocytes across all the treatment groups. The higher PB extrusion rates observed in VitroMat-protect^TM^ and BO-IVM^TM^ may be attributed to specific factors in these media that better support oocyte maturation post-cryopreservation. Cryopreservation can induce stress and cause damage to oocytes, making it more difficult for them to complete meiotic division efficiently [33,34]. Thus, media supporting oocyte recovery and maturation post-cryopreservation may help mitigate the detrimental effects of freezing and thawing, enhancing meiotic progression and PB extrusion.

Assessment of oocyte morphology is a difficult task since underlying mechanisms that change the appearance of oocytes are multifactorial and complex, particularly in vitrified oocytes. The oocyte survival rate was affected by the following analyzed oocyte abnormalities: FPBs, LVs, DOs, and CC. First, the PB morphology of cattle oocytes can indicate further embryo development and viability. This study’s results demonstrated higher chances of FPBs being seen in vitrified oocytes matured in Vitromat-Protect^TM^ and BO-IVM^TM^ compared to TCM199. Oocytes with FPBs during GRS denudation may still have impaired cytoplasmic competence. Several studies [40,41] have found that FPBs have a decreased potential for fertilization and impaired development. Fancsovits et al. [42] recommend against transferring them and cryopreserving them. Therefore, other programs need to assess FPBs prospectively to establish a consensus on their role in an IVM setting. This study demonstrated that LVs, DG, and cracked CC rates significantly increased vitrified rather than non-vitrified oocytes in all treatment groups. This may prove that oocyte cryopreservation causes some irreversible structural damage. Lasiene et al. [43] reported the quality of oocytes as assessed by the structure of COCs. This procedure gives us some information about the quality of oocytes [43]. Adding to that, the results of this study provide valuable insights into the effects of vitrification and different maturation media on the developmental competence of vitrified oocytes, particularly in terms of COCs’ morphometry, morphology, and maturation assessment.

## 5. Conclusions

Altogether, the cryopreservation process affects the GRS in both vitrified immature and mature oocytes, with the most significant reduction seen in mature oocytes, which could affect the developmental competence and viability of oocytes post-thawing. However, it does not affect the structural integrity of ZP in non-vitrified and vitrified oocytes. Additionally, vitrified oocytes matured in VitroMat-protect^TM^ and BO-IVM^TM^ showed better maturation outcomes than those matured in TCM199. Vitrification influenced oocyte quality, with a higher incidence of FPB and CC in vitrified oocytes matured in Vitromat-Protect^TM^ and BO-IVM^TM^ than TCM199.

## 6. Recommendation

To improve oocyte quality and developmental potential in assisted reproduction, the selection of vitrification solutions, warming procedures, and maturation media conditions should be optimized to reduce oocyte structural damage. The differentiation of the ability of vitrification solutions and maturation media to support in vitro development, along with their ability to yield viable embryos, may be necessary for better outcomes. Protocol choice should be adapted to the specific situation, and further research is needed to improve embryo production from vitrified oocytes.

## Figures and Tables

**Table 1:** Composition ART Lab Solutions vitrification and warming media (according to the manufacturers’ leaflets).

Solution	ART Lab Solutions™-Vitrification and Warming	
	Composition	Time
Handling media	Buffered medium + bovine serum albumin	5–10 min
Vitrification solution 1	Buffered medium + bovine serum albumin + ethylene glycol dimethylsulfoxide	3 min
Vitrification solution 2	Sucrose solution + ethylene glycol + dimethylsulfoxide	30 s
Warm 1	Handling media + 1 M sucrose solution	<1 min
Warm 2	Handling media + 1 M sucrose solution	5 min
Warm 3	Handling media + 1 M sucrose solution	5 min
Warm 4	Handling media	1–5 min

**Table 2:** Composition of standard tissue culture vitrification and warming medium (ARC lab prepared media).

Solution	TCM 199-Vitrification and Warming	
	Composition	Time
Base (BS)	TCM199 + fetal bovine serum	1 min
Rinsing	Base + fetal bovine serum	3 min
Holding medium	Base + fetal bovine serum + dimethylsulfoxide + ethylene glycol	3 min
Vitrification solution	TCM199 + fetal bovine serum + ethylene glycol + dimethylsulfoxide + sucrose	30 s
Warming	Dulbecco’s phosphate-buffered saline + fetal bovine serum + sucrose	30 s
Rehydration	Dulbecco’s phosphate-buffered saline + fetal bovine serum + sucrose	1 min

**Table 3:** Composition of BO vitrification and warming medium (according to the manufacturers’ leaflets).

Solution	BO-VitriCool^TM^ and BO-VitriWarm^TM^	
	Composition	Time
Pre-incubation	Ethylene glycol + dimethylsulfoxide-based formula (serum-free)	2 min
Cooling 1	Ethylene glycol + dimethylsulfoxide-based formula	2 min
Cooling 2	Ethylene glycol + dimethylsulfoxide-based formula	30 s
Warming 1	Sucrose + albumin (serum free)	3 min
Warming 2	Sucrose + albumin	2 min
Warming 3	Sucrose + albumin	2 min
Warming 4	Sucrose + albumin	1 min

**Table 4:** Comparison of maturation capacity of cattle vitrified oocytes in different maturation media.

Treatment	Type of the Oocyte (*n* = 900)	COC Expansion	PB%
Vitromat-Protect^TM^	Non-vitrified	93.30 ± 6.96 ^a^	67.30 ± 5.67 ^a^
	Vitrified	34.00 ± 11.02 ^c^	24.60 ± 11.93 ^bc^
BO-IVM^TM^	Non-vitrified	94.75 ± 6.70 ^a^	71.50 ± 12.76 ^a^
	Vitrified	52.28 ± 7.06 ^b^	35.14 ± 5.01 ^b^
TCM199	Non-vitrified	87.33 ± 13.08 ^a^	61.90 ± 7.79 ^a^
	Vitrified	28.70 ± 10.03 ^c^	18.44 ± 8.00 ^c^

^a–c^ Values within the same column with different superscripts differ significantly (*p* < 0.05). COCs = cumulus oophorus complexes, and PB = polar body.

**Table 5:** Metaphase II oocyte morphological comparisons between vitrified and non-vitrified oocytes.

Treatment	Type of the Oocyte	FPB	LV	DG	CC
Vitromat-Protect^TM^	Non-vitrified	2.00 ± 4.47 ^c^	15.30 ± 8.44 ^abc^	2.00 ± 4.47 ^cd^	0.00 ± 0.00 ^d^
	Vitrified	19.30 ± 3.97 ^a^	22.60 ± 6.51 ^a^	15.80 ± 8.24 ^a^	24.50 ± 10.53 ^a^
BO-IVM^TM^	Non-vitrified	1.75 ± 3.52 ^c^	10.00 ± 3.46 ^c^	0.00 ± 0.00 ^d^	0.00 ± 0.00 ^d^
	Vitrified	16.14 ± 5.20 ^ab^	24.00 ± 5.50 ^a^	15.14 ± 9.38 ^ab^	31.42 ± 7.32 ^a^
TCM199	Non-vitrified	0.70 ± 2.21 ^c^	12.00 ± 7.95 ^bc^	1.40 ± 2.95 ^d^	0.00 ± 0.00 ^d^
	Vitrified	13.90 ± 4.54 ^b^	20.70 ± 8.01 ^ab^	16.00 ± 6.44 ^a^	18.70 ± 7.04 ^bc^

^a–d^ Values within the same column with different superscripts differ significantly (*p* < 0.05). FPB = fragmented polar body, LV = large vacuole, DG = degenerated, and CC = cracked cytoplasm.

## Data Availability

The data are available from the corresponding author.
